# Ohio State Veterinary Medical Association

**Published:** 1891-02

**Authors:** Wm. H. Gribble

**Affiliations:** Secretary


*Ohio State Veterinary Medical Association.*—The eighth annual meeting of the Ohio State Veterinary Medical Association was held in Wells Post Hall, Columbus, January 14, 1891. The meeting was called to order at 2 P.M., with President Geo. W. Butler in the chair. The following members were in attendence: J. S. Butler, T. B. Cotton, J. Charlesworth, W. F. Derr, W. C. Fair, T. B. Hillock, W. R. Howe, W. A. Labron, A. H. Logan, J. M. Waddell, T. Kerr, Walter Shaw, W. H. Gribble, W. Torrence, J. D. Fair, N. C. McLean, C. Christman, E. H. Shepherd, E. C. Barnett, and also Drs. Jones, King, and Brety and Prof. Townsend.

The minutes of the previous meeting were read and approved. Dr. Geo. W. Butler, the president, then rendered his annual address in a novel and somewhat poetical manner.

Nomination and election of officers followed which resulted as follows: President, W. R. Howe, Dayton; 1st Vice-President, E. H. Shepherd, Cleveland; 2d Vice-President, J. W. Fair, Berlin; 3d Vice-President, M. C. McLean, Jeromeville; Secretary, W. H. Gribble, Washington, C. H.; Treasurer, T. B. Hillock, Columbus. The following graduates were proposed for membership: Dr. Neil Jones, of Chilicoth, vouched for by Drs. Shaw and Butler; Dr. F. J. King, vouchers, Drs. Butler and Howe; Dr. S. Bretz, vouchers Drs. Cotton and Hillock. A ballot was taken, and all three were declared elected. Each new member in turn was called upon for an address, and responded in a few brief remarks

Correspondence. A letter from Prof. Liautard stating his regards for the Ohio society and offering to print minutes of meeting. A vote of thanks were then given Dr. Liautard.

The secretary next read a communication from Dr. Kinsman, Sec'y of the Ohio State Live Stock Com., in response to Drs. Butler's vote of censure passed at the July meeting. The communication showed that the state commission did not have so much authority vested in it as had been presumed and denied that the commission had been negligent in its sworn duties. The communication was well received until the final paragraphs were reached when Dr. Kinsman took occasion to slur the Veterinary profession of Ohio, on the ground of too much disagreement, this slur being entirely uncalled for, as the profession of which Dr. Kinsman is an honored member, can certainly not boast of agreeing on diagnosis in every case; especially is this shown in suits at law for malpractice. A heated discussion followed in which Drs. Fair, Butler, Cotton, Shaw, Hillock, and Gribble took part. Moved, by Dr. Gribble, seconded by Dr. Wight, that this discussion be postponed until later and that Dr. Kinsman be especially asked to be present. Carried. Dr. Fair urged hearty co-operation between the Veterinary profession and the Live Stock profession and the Live Stock Commissions. He could not say he had been treated with justice, but he desired unity. Dr. Cotton gave his experience with the commission. Dr. Gribble said they had been very prompt in aiding him in an outbreak of Glanders.

Dr. J. C. Meyers, Sr. then read a paper, "Tracheotomy and Laryngeal Injections in Affections of the Throat." He exhibited a tracheotomy tube to be used in cases where it was expected that it would not be needed for more than a few days; the tube is in the form of a trocar and canula and is passed through the trachea transversely and retained in place by set screws. The paper was received with applause. Dr. J. S. Butler asked if such injections would not benefit chronic coughs. Dr. Meyers cannot see why not if sufficient care be used to wash the mucous membrane.

Dr. Newton described a case of absesses following use of tracheotomy tube, with fatal termination. Dr. Meyers thought the absesses to be the result of false membranes, &c.; others that the case was irregular strangles. Dr. Charleswort reported a case where constriction or rather a flattening of the trachea followed the use of a tube, it was operated on twice to relieve the complication and at last was cured by use of a long, round silver tube being kept in the trachea until it adjusted itself to the shape of the tube. Dr. Fair said less trouble would result if none of the rings of the trachea were completely severed, he removes about half of two different rings.

Dr. Butler read from the London *Journal* a report of a case where the epiglottis had become misplaced and held by the *velum palati*.

Dr. Shepherd read an essay on "Hypo Sulphite of Soda." It elicited considerable discussion especially as regards its use in skin diseases and gastric fermentation. Dr. Torrence gave his experience in treating these troubles with Chloro-naptholum, stating this latter drug to be a deodorizer and disinfectant. A discussion on the treatment of Azoturia was taken up by Dr. Wight, Meyer, Derr, Newton and others, which showed the variable fatality in different districts. Some members expected when the animal was prostrated it was as good as dead. Dr. Derr had a case to which he had called Dr. Wight and others where paralysis followed the disease and where after nine months treatment the case recovered and is working to-day, but covered with scars from bed sores.

Meeting adjourned at 6 P. M.

*Evening Session.*

Meeting called to order with Dr. Shepherd in the chair.

An essay by Dr. Cotton was read on remedies for Parturient Apoplexy. His pet prescription is ;

℞

Spts. Ammon. arom. ℥ x

Spts. Ætherus Nii. ℥ xx

Sig. ℥iii every half hour for five hours ; or every one hour for five hours.

He also gives Magnes. Sulp. ℥xxiv. Large doses of stimulants were, in his opinion, curative, to be followed with Nux Vomica, if necessary, and injections. Drs. Shaw, Butler, Torrence, Derr, Newton, and Townsend lauded Oleum Tiglii, even when deglutition was paralyzed.

Place for the next meeting was now discussed. Moved by Dr. Torrence, seconded by Dr. Hillock, that the President, Secretary, and Dr. Newton correspond with the officers of the Michigan State association and mutually agree to meet with them at some place and time during the summer. Carried.

Committee on contagious diseases rendered report. Dr. Hillock was of opinion that actinomycosis is contagious and cited several cases in proof.

Drs. Shepherd, Torrence and Fair, gave description of an outbreak of Stomititis in Cleveland. Dr. J. D. Fair believed in a volitile virus in Glanders.

Dr. Gribble could not believe in such a virus, had had experience with the disease and was well satisfied that it resulted from inoculation, in fact had never seen a case wherein there was a particle of proof to sustain spontaneous origin. If he believed as Dr. Fair, Ohio would have one less veterinary surgeon in its employ for fear of infection.

Dr. Fair presumed no one would be a week making the examination. His idea was not championed by any other member present.

Dr. Howe reported several cases of death on one farm. Animals would be dumpish, inclined to stay in one place, temperature and pulse about normal, from examination of them one would not think the animals sick, but their facial expression showed otherwise, there was some paralysis, groom claimed inability to swallow, youngest died first, old horses lived several days. Post Mortem (gross) revealed nothing to satisfy him.

Dr. Hillock suggested diphtheria.

Dr. Fair corn stalk disease.

Dr. Buter thought the fact of diphtheria was not sustained in the horse.

Dr. Hillock said the dogs and cats of Columbus are now having an epidemic of diphtheria.

A discussion as to asking veterinary legislation was entered into, but finally decided to be let alone as it was useless to expect any help.

Dr. J. S. Butler stated he had sold his practice and was about to move west, and asked to withdraw from the association. Dr. Newton moved and Dr. Torrence seconded that Dr. J. S. Butler be elected an honorary member of this association. Carried.

Dr. Gribble offered amendment to constitution, viz., that hereafter officers do not take their respective offices until at the close of the meeting at which they are elected.

Bills were allowed, and Treasurer reported about $300,00 in treasury.

The association adjourned to meet with Michigan society on the order of the President and call by the Secretary.

WM. H. GRIBBLE, *Secretary.*

